# A Piezoresistive Tactile Sensor for a Large Area Employing Neural Network

**DOI:** 10.3390/s19010027

**Published:** 2018-12-21

**Authors:** Youzhi Zhang, Jinhua Ye, Zhengkang Lin, Shuheng Huang, Haomiao Wang, Haibin Wu

**Affiliations:** School of Mechanical Engineering and Automation, Fuzhou University, Fuzhou 350116, China; zhangyouzhi606@gmail.com (Y.Z.); yejinhua@fzu.edu.cn (J.Y.); 15396116230lzk@gmail.com (Z.L.); shuhenghuang.cn@gmail.com (S.H.); wanghaomiao.cn@gmail.com (H.W.)

**Keywords:** electronic skin, contact position detection, contact force detection, RBF neural network

## Abstract

Electronic skin is an important means through which robots can obtain external information. A novel flexible tactile sensor capable of simultaneously detecting the contact position and force was proposed in this paper. The tactile sensor had a three-layer structure. The upper layer was a specially designed conductive film based on indium-tin oxide polyethylene terephthalate (ITO-PET), which could be used for detecting contact position. The intermediate layer was a piezoresistive film used as the force-sensitive element. The lower layer was made of fully conductive material such as aluminum foil and was used only for signal output. In order to solve the inconsistencies and nonlinearity of the piezoresistive properties for large areas, a Radial Basis Function (RBF) neural network was used. This includes input, hidden, and output layers. The input layer has three nodes representing position coordinates, *X*, *Y*, and resistor, *R*. The output layer has one node representing force, *F*. A sensor sample was fabricated and experiments of contact position and force detection were performed on the sample. The results showed that the principal function of the tactile sensor was feasible. The sensor sample exhibited good consistency and linearity. The tactile sensor has only five lead wires and can provide the information support necessary for safe human—computer interactions.

## 1. Introduction

Robotic intelligent technology is booming all over the world, and the tactile sensor is one of the prerequisites for achieving robot intelligence. The tactile sensor, one of the most important wearable devices, is directly related to the intellectualization of the next generation of robots, medical devices, and human prosthesis technologies [1,2,3]. It has become one of the hottest topics in academic research [4,5,6].

Based on a variety of transduction principles, such as piezoresistive [7], capacitive [8], piezoelectric [9], electromagnetic [10], and optoelectronic principles [11], etc., researchers have developed different types of tactile sensors capable of detecting one or more kinds of tactile information such as contact position, force, relative sliding, temperature, and roughness. Normally, the parts of the tactile sensor can be divided into those of array structure and the non-array structure. The array tactile sensor integrates a certain number of sensor units, which constructs the detection area. It is mostly designed and manufactured based on micro-electro-mechanical system (MEMS) technology and presents a small structural size, good sensitivity, and high accuracy. For instance, Takao et al. [12] designed a flexible pressure sensor matrix with organic field-effect transistors, and the size of each sensor cell was only 2 mm. The sensor had good flexibility and high spatial resolution. Ahmed et al. [13] developed a magnetic nanocomposite cilia tactile sensor. The sensor could detect pressure as low as 3.5 mN and had an ultrahigh sensitivity. However, the array tactile sensor also has some defects. Normally, the detection is discontinuous. The sensing signals must be acquired by scanning each of the sensing cells, so the spatial resolution and the response time of the sensor are often difficult to balance. In addition, the amount of data needing to be processed by the sensor will be enormous for large area use.

The non-array tactile sensor is capable of using a single sensing unit to detect contact information and can be flexibly attached to the surface of the robots. For instance, Dana et al. [14] proposed an artificial skin that detected the position and speed of a slipping object using a single force sensor. The sensor had an average speed sensing resolution of 10 mm/s, an average position sensing resolution of 15 mm, and was robust in its calculation of various grip conditions. Visentin et al. [15] proposed a deformable tactile sensor for continuous sensing based on electrical impedance tomography (EIT). The sensor was made of conductive fabric and can be used for multi-point contact position detection. Compared with the array tactile sensor, the non-array tactile sensor does not need to scan the sensing cells point by point to obtain sensing signals and has fewer lead wires. Thus, the non-array tactile sensor is more suitable for the “electronic skin” of robots.

Tactile sensors, as the “electronic skin” of robots, usually are used to flexibly cover relatively large areas of the robot’s surface. Contact position and force are the most important kinds of tactile information for human—robot interaction. At present, researchers have developed some non-array tactile sensors for detecting contact position or force. For instance, Pan et al. [16] proposed a flexible tactile sensor fabricated with conductive fabric that could detect contact position. The sensor could be used to detect contact position over a large area. There were fewer leading wires, and the signal processing was simple. However, the position detection capability of the sensor was still discrete. Wu et al. [17] developed a non-array tactile sensor based on a distributed planar electric field. The sensor was capable of continuous position detection and had good flexibility and simple signal processing, but it could only detect single-point contact. David et al. [18] proposed a tactile sensor fabricated with conductive fabric based on electrical impedance tomography. The sensor could be made into different shapes and used for multi-point contact position detection. Zhang et al. [19] developed a low-cost touch sensing sensor using electric field tomography. The sensor could be integrated onto the surface of an object by spraying so that it could even be used to detect contact positions on the surfaces of 3D objects. However, the EIT and similar technologies require complex mathematical algorithms to obtain the detecting information. It thus needed more electrodes and better computing capacity of the hardware, and its practicability was subject to such restrictions. In addition, Markus et al. [20] proposed a tactile sensor based on piezoresistive conductive rubber. The sensor was made of piezoresistive conductive rubber and copper wire. The copper wire was sewn into the rubber to form a staggered grid, and elastic foam was attached to the surface of the sensor. The sensor was capable of detecting the contact position and contact force. However, it still contained many wires, and the wiring was complicated. Wu et al. [21] designed a tactile sensor based on the resistive and capacitive sensing method. The sensor had a five-layer structure and only six lead wires. It was capable of detecting both the collision position and force, but sensitivity to force detection would decrease with the increase of the sensor’s size.

In this paper, we propose a novel non-array thin-film tactile sensor, which can be used for detecting the contact position and force. The sensor has a multi-layer structure and only five lead wires. The sensor has good flexibility, low-cost, favorable robustness, and can be used for full-body robot clothing.

## 2. Development of the Tactile Sensor

### 2.1. Structure of the Tactile Sensor

**Figure 1:**
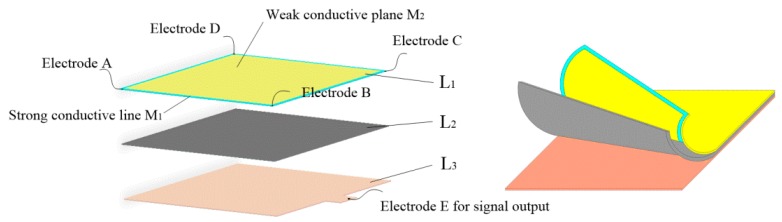
The structural model of the tactile sensor.

The tactile sensor is a “sandwich” structure with three layers, labeled by L_1_–L_3_, respectively, as shown in Figure 1. L_1_ is the conductive plane designed according to a special structure. It is composed of the strong conductive line, M_1_, and a weak conductive plane, M_2_. M_1_ is coated around M_2_, and the conductivity of M_1_ is much higher than that of M_2_. There are four electrodes fitted at four corners of M_1_. When a bias excitation is applied to its two diagonal electrodes, a uniform electric field will be generated on the weak conductive plane. L_2_ is the force sensing element, that is, the piezoresistive film. Its internal resistance decreases with the increase of external pressure. L_3_ is the conductive plane with good conductivity, and the whole layer of L_3_ is used as signal output and is referred to as electrode E. There is a seamless fit between L_2_ and L_3_, and a small amount of gas is filled and sealed between L_1_ and L_2_. When the electronic skin is not stressed, there is no contact between L_1_ and L_2_.

The single detecting process of the sensor consists of three stages when one point is pressed. In the first stage, a bias excitation is applied to electrodes A and C of the sensor, and a uniform electric field will be generated on the weak conductive plane of L_1_. L_1_ will be deformed and will have contact with L_2_ and L_3_ at the pressed point. Next, the potential of the pressed point on the weak conductive plane of L_1_ is extracted by L_3_. According to the linear relationship between position and potential in the uniform electric field, the coordinate of the contact point can be easily calculated. In the second stage, a bias excitation is applied to electrodes B and D of the sensor, and a uniform electric field orthogonal to the previous electric field will be generated on the weak conductive plane of L_1_. Similarly, it is easy to calculate the coordinate of a pressed point by extracting its potential. The positions and coordinates of any pressed point can therefore be detected. In the third stage, because L_1_ is deformed and in contact with L_2_ and L_3_ at the pressed point, the sensor is able to calculate the contact force by extracting the resistance between electrodes C and E based on the piezoresistive properties of the force sensing element.

Thus, the tactile sensor only uses a single sensing unit to detect contact position and force.

### 2.2. Contact Position Detection

The key to position detection is to alternately construct two uniform electric fields in different directions on the weak conductive plane of L_1_. In this paper, L_1_ is composed of the strong conductive line M_1_ and the weak conductive plane M_2_. M_1_ is coated around M_2_, and the conductivity of M_1_ is much higher than that of M_2_.

**Figure 2:**
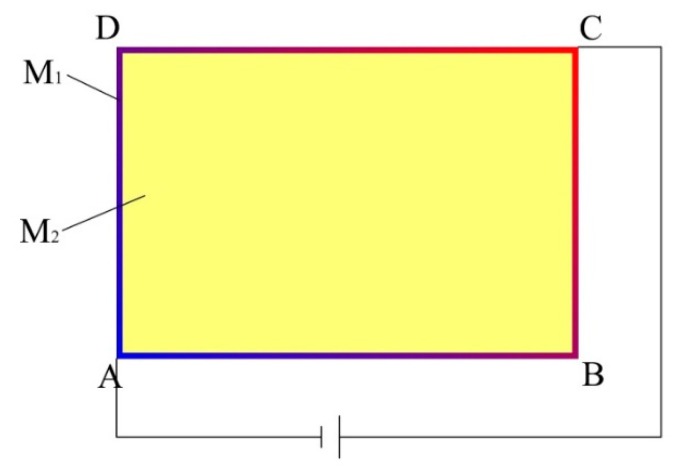
The working schematic diagram of L_1_.

As shown in Figure 2, a bias voltage is applied to the two diagonal points A and C of M_1_, and since the conductivity of M_1_ is much greater than that of M_2_, most of the current will flow from C to A in M_1_. A potential distribution of the linear increase will be generated from A to C in M_1_. Since M_1_ has good contact with M_2_, the potential distribution of the linear increase will also be generated from A to C on the boundary of M_2_, that is the boundary potential distribution necessitated by constructing a uniform electric field.

Suppose M_2_ is a homogeneous conducting plane, its internal potential distribution φ(x,y) satisfies the Laplasse equation:

(1) $$\frac{\partial^{2}\varphi }{\partial^{2}x}+\frac{\partial^{2}\varphi }{\partial^{2}y}=0.$$

**Figure 3:**
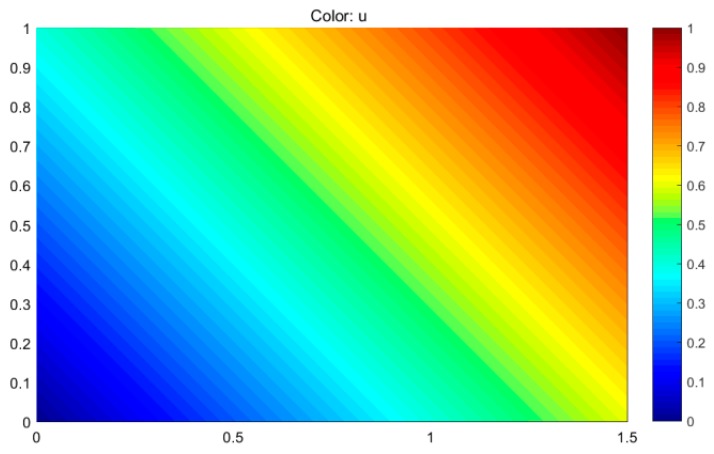
The potential distribution of the rectangular homogeneous conductive plane with the boundary potential distribution of linear increase.

Using the PDE toolbox of MATLAB, the model of the rectangular homogeneous conductive plane with the boundary potential distribution of linear increase shown in Figure 2 is established. Next, the potential distribution of the model is solved, as shown in Figure 3.

**Figure 4:**
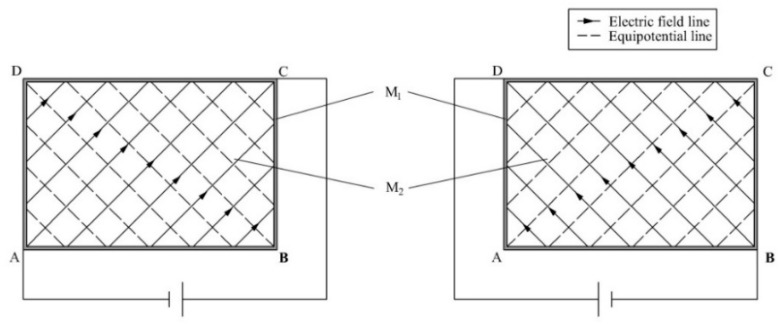
The distribution of the electric field and potential in M_2_ of L_1_.

As shown in Figure 3, a uniform electric field is generated along the 45° direction in the rectangular homogeneous conductive plane. Therefore, when a bias voltage is applied to the two diagonal points A and C of M_1_ of L_1_, a uniform electric field will be generated along the 45° direction in M_2_ of L_1_. Similarly, when a bias voltage is applied to the two diagonal points B and D of M_1_ of L_1_, a uniform electric field will also be generated along the 135° direction in M_2_ of L_1_, as shown in Figure 4.

**Figure 5:**
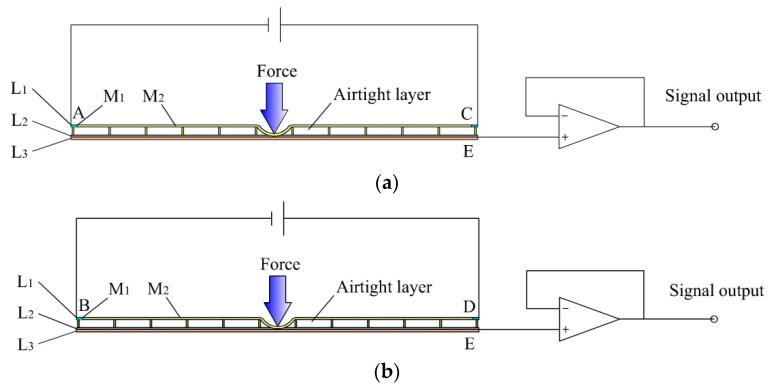
Position detection. (**a**) Position detection in the *X*-axis. (**b**) Position detection in the *Y*-axis.

The potential of any point in M_2_ of L_1_ can be detected as shown in Figure 5. Firstly, a constant DC bias voltage is applied to the two diagonal points A and C in M_1_ of L_1_, and a uniform electric field will be generated in M_2_ of L_1_. If an external force is applied to the surface of the sensor, L_1_ will deform, pass through the airtight layer and contact L_2_ and L_3_ at the contact point. Next, the potential of the whole layer of L_3_ will be equal to the potential of the contact point in M_2_ of L_1_. L_3_ is connected to an operational amplifier with infinite input impedance so that it is able to output the potential of the contact point. Similarly, if a constant DC bias voltage is applied to the two diagonal points B and D in M_2_ of L_1_, the potential of the contact point can also be obtained from L_3_. According to the linear relationship between the position and the potential in the uniform electric field, the *X* and *Y* coordinates of any contact point in M_2_ of L_1_ can be easily calculated. Thus, the detection of the contact position can be realized.

### 2.3. Contact Force Detection

**Figure 6:**
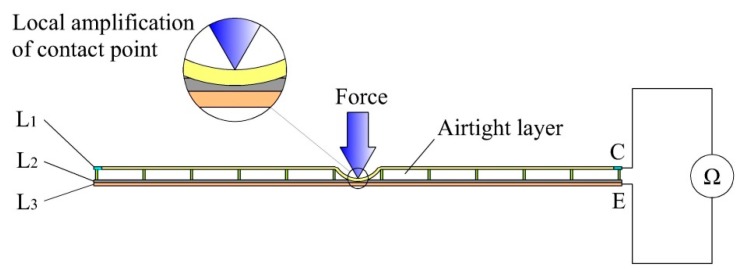
The schematic diagram of contact force detection.

The principle functioning of contact force detection of the sensor is based on the piezoresistive effect of the force sensing element. The whole layer of L_2_ is made of piezoresistive film and used as the force sensing element of the sensor. As shown in Figure 6, when an external force is applied to the surface of the sensor, L_2_ is pressed by L_1_ and L_3_ at the contact point. The resistance between L_1_ and L_3_ of the sensor will then decrease with the increase of the external force. Therefore, the contact force can be detected by extracting the resistance between electrode C and electrode E of the sensor.

It is worth mentioning that there is an airtight layer between L_1_ and L_2_ of the sensor. This makes the resistance between L_1_ and L_3_ very high when the sensor is not pressed by an external force. Thus, the power consumption of the sensor can be effectively reduced.

**Figure 7:**
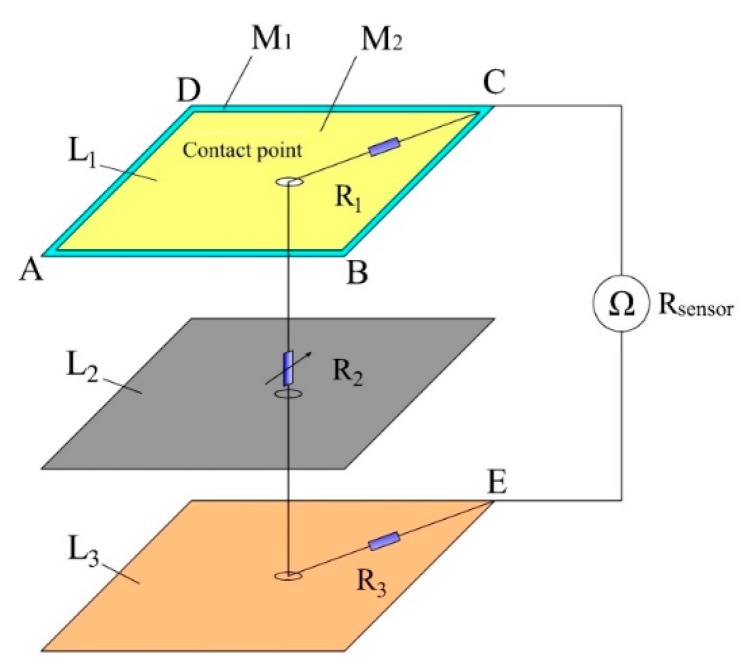
The resistance model for detecting contact force.

The resistance model for detecting contact force is established as shown in Figure 7. When the sensor is pressed by an external force, the total resistance $R_{\mathrm{sensor}}$ between electrodes C and E of the sensor can be expressed as follows:

(2) $$R_{\mathrm{sensor}}=R_{1}+R_{2}+R_{3}$$

where $R_{1}$ is the resistance between the contact point in M_2_ of L_1_ and electrode C, which is related to the position of the contact point. $R_{2}$ is the resistance between L_1_ and L_3_ at the contact point. Ignoring the contact resistance, $R_{2}$ can be regarded as the resistance of the piezoresistive film at the contact point, which decreases with the increase of the external force. $R_{3}$ is the resistance between the contact point in L_3_ and electrode E. Since L_3_ has good conductivity, $R_{3}$ is very small and negligible.

**Figure 8:**
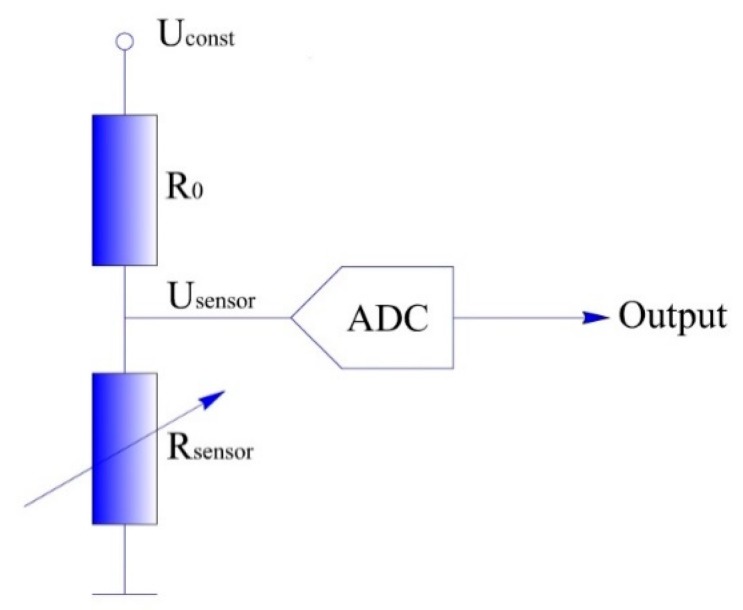
The schematic diagram of the contact force detection circuit.

The detecting circuit of the contact force can be realized by a simple resistor divider circuit, as shown in Figure 8. The sensor (represented as $R_{\mathrm{sensor}}$ in Figure 8) is connected in series with the fixed resistance $R_{0}$, and a constant voltage $U_{\mathrm{const}}$ is applied at both ends of the circuit; then the output voltage $U_{\mathrm{sensor}}$ is:

(3) $$U_{\mathrm{sensor}}=\frac{U_{\mathrm{const}}\cdot R_{\mathrm{sensor}}}{R_{0}+R_{\mathrm{sensor}}}.$$

Finally, $U_{\mathrm{sensor}}$ is transmitted to the microcontroller by the A/D conversion so that the contact force can be detected.

### 2.4. Sample Fabrication

**Figure 9:**
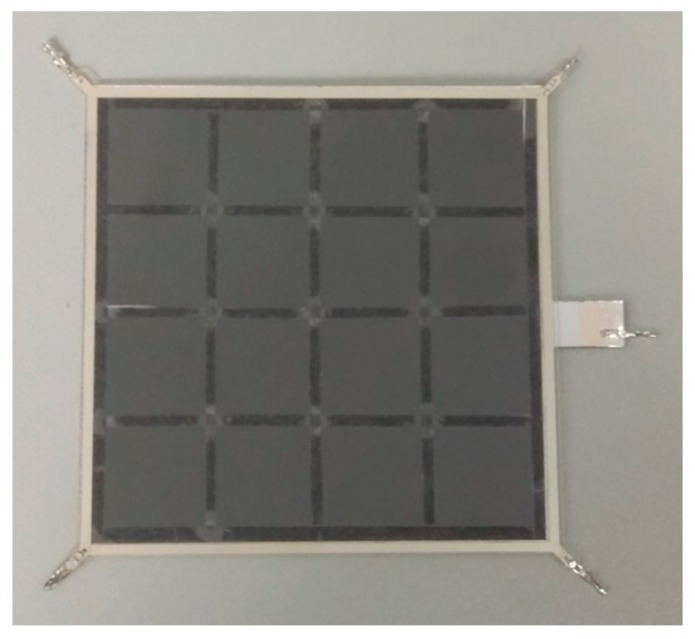
The sensor sample.

**Figure 10:**
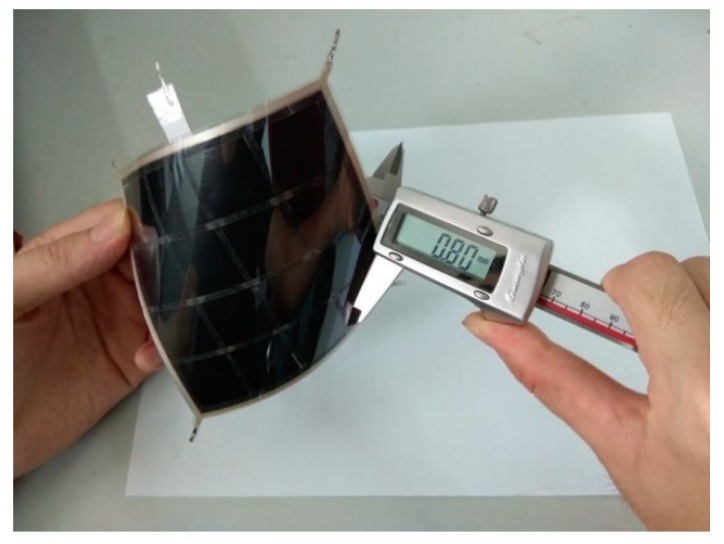
The thickness of the sensor sample.

In order to further verify the feasibility of the tactile sensor, a sample of the sensor was fabricated. Conductive silver paste was used as the strong conductive line, M_1_ of L_1_, and the conductive indium-tin oxide polyethylene terephthalate (ITO-PET) film was used as the weak conductive plane, M_2_ of L_1_. Velostat is a conductive film made of polyolefins foil impregnated with carbon black, produced by the 3M company. It has favorable piezoresistive properties. Therefore, the Velostat film was used as the force sensing element of the sensor. The detailed fabrication process is as follows:

(1) Upper layer L_1_: the ITO-PET film with sheet a resistance of 100 Ω/cm^2^ and the thickness of 0.175 mm (where the thickness of the ITO film is 70 nm) was cut to a square with 100 × 100 mm. The conductive silver paste with a conductivity of about 2.4 × 10^7^ S/m was printed with screen printing on the ITO-PET film. Four aluminum electrodes were fitted onto the four corners of the rectangular circuit printed with conductive silver paste.
(2) Middle layer L_2_: The Velostat film with a thickness of 0.1 mm was cut into a square with 100 × 100 mm. A double-sided glued grid was used to bond layers L_1_ and L_2_ together to form an airtight isolation layer. The grid had a thickness of 0.3 mm and mesh size of 25 × 25 mm.
(3) Lower layer L_3_: The aluminum foil with good conductivity was cut into a square of 100 × 100 mm dimensions. A PET film was attached to one side of the aluminum foil as an insulating protective layer. An electrode was fitted onto the edge of the aluminum foil for signal output.
(4) Assembling: The 3M-9495# double-sided adhesive was attached to the edge of the lower layer, and the upper, middle, and lower layers were aligned and closely bonded. The sensor sample is shown in Figure 9 and Figure 10.

### 2.5. Piezoresistive Properties of the Velostat Film

The sensor detects the contact force based on the piezoresistive properties of the force sensing element. As shown in Figure 9, the sensor used a large piece of Velostat film as the force sensing element. Ideally, the piezoresistive properties of all regions of the Velostat film should be consistent. Then, the mapping relationship between force and resistance can be tested. According to the mapping relationship, the contact force can be easily calculated. Therefore, it is necessary to further study the consistency of the piezoresistive properties of the Velostat film.

**Figure 11:**
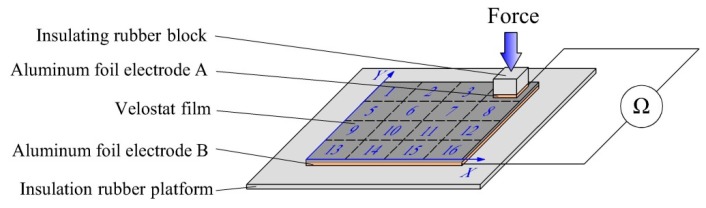
Test device for testing the consistency of the piezoresistive properties of the Velostat film.

**Figure 12:**
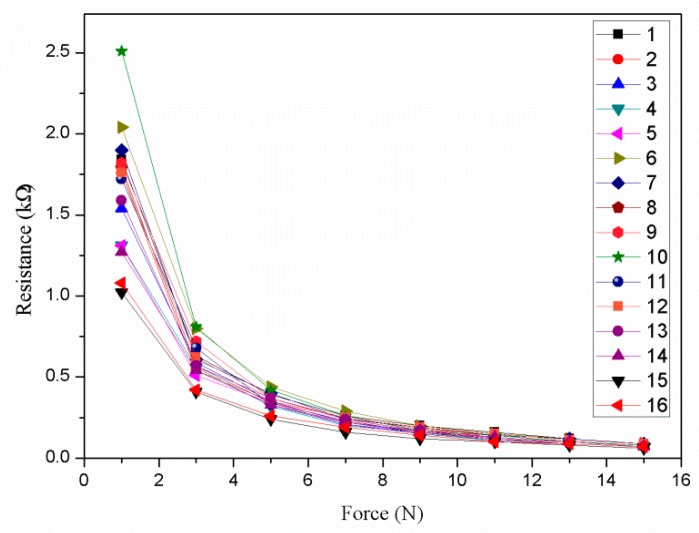
The mapping relationship between the force and resistance of the sixteen small squares of the Velostat film.

A simple test device was designed to test the consistency of the piezoresistive properties of the Velostat film. The test device is shown as Figure 11. First, a piece of the Velostat film with 100 × 100 mm dimensions was divided into sixteen small squares of 25 × 25 mm size, and two pieces of aluminum foil were used as electrodes to clamp the Velostat film. Then, a force of 1–15 N was applied to them, respectively, and the resistances between the two electrodes were recorded by a multimeter. Two rubber blocks were used to insulate the aluminum foils from the outside. The size of the aluminum foil electrode A was 15 × 15 mm, and the size of the aluminum foil electrode B was 100 × 100 mm. The mapping relationship between the force and resistance of the sixteen small squares of the Velostat film is shown in Figure 12.

As shown in Figure 12, there are sixteen piezoresistive properties curves, which are obviously different. Therefore, the piezoresistive properties of the sixteen small squares of the Velostat film are inconsistent. The main reasons for the differences are that the distribution of conductive particles in the interior of the Velostat film is uneven and the contact resistance between the Velostat and aluminum foil of different small squares are different.

Therefore, if the average of the sixteen piezoresistive properties curves is used to calibrate the sensor for detecting the contact force, it will generate a large error of the contact force detection. In addition, the resistance $R_{1}$, shown in Figure 7, is also related to the position of the contact point. In summary, the contact force detection of the sensor is closely related to the position of the contact point. In order to solve this problem, on the premise of obtaining the position of the contact point, the Radial Basis Function (RBF) neural network algorithm was used to train and calibrate the tactile sensor for detecting the contact force.

## 3. Detection Algorithm Using RBF Neural Network

### 3.1. RBF Neural Network

Artificial Neural Networks (ANN) have been a hotspot in the field of artificial intelligence and have been widely applied for pattern recognition, intelligent robots, automatic controls, biology, medicine, economics, etc. Some papers on tactile sensors using a neural network for texture recognition [22], contact shape recognition [23], three-dimensional force decoupling [24,25], etc., have been reported. For instance, Ken et al. [26] developed a neurorobotic texture classifier with a recurrent spiking neural network which could classify surface textures through touch. Anany et al. [27] designed a soft magnetic 3-D force sensor. The sensor was a pyramid-shaped tactile unit featuring a tri-axis Hall element and a magnet embedded in a silicone rubber substrate. The non-linear mapping between the 3-D force vector and the Hall effect voltages was characterized by training a neural network. In this paper, the RBF neural network was used as a contact force detection algorithm. The mapping between the output resistance, contact position, and the applied force was established by training the RBF neural network.

**Figure 13:**
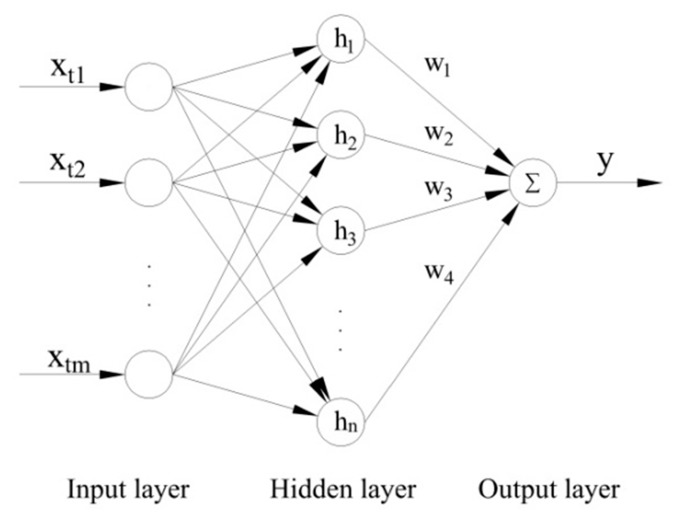
The schematic diagram of the Radial Basis Function (RBF) neural network.

The structure of the Radial Basis Function (RBF) neural network is shown in Figure 13. The RBF neural network is a three-layer feedforward network with a single hidden layer. It consists of an input layer, a hidden layer, and an output layer. The hidden layer can transform the input vectors of the input layer and map the input sample data of the low-dimensional mode into the high-dimensional space. The nodes of the hidden layer are then weighted. Finally, the sums of the weighted nodes are output by the output layer. Therefore, the RBF neural network has the abilities necessary for mapping a nonlinear separable space into a linearly-separable space.

The hidden layer is a set of radial basis functions. The commonly used radial basis function is the Gauss function:

(4) $$h_{i}=\exp\left[-\frac{\left\|x_{t}-c_{i}\right\|}{2\sigma_{i}^{2}}\right]\left(i=1,2,\cdots ,n\right)$$

where $x_{t}={\left(x_{t1},x_{t2},\cdots ,x_{tm}\right)}^{T},t=1,2,\cdots ,n$, is the *t*th input vector of the input layer, and n is the total number of the input vectors, $c_{i}$ is the center of the *i*th radial basis function, $\sigma_{i}$ is the extended constant of the center of the *i*th radial basis function, which determines the width of the basis function around the center point, $c_{i}$, $\left\|x_{t}-c_{i}\right\|$ is the norm of the vector $x_{t}-c_{i}$, representing the distance between $x_{t}$ and $c_{i}$.

Thus, the mapping function from the input layer nodes to the output layer nodes can be expressed as:

(5) $$y_{t}=\sum_{i=1}^{n}w_{i}\exp(-\frac{1}{2\sigma^{2}}\left\|x_{t}-c_{i}\right\|)$$

where $w_{i}$ is the weight of the hidden layer mapping to the output layer, and $y_{t}$ is the output vector of the output layer corresponding to the input vector, $x_{t}$.

Let $Y_{t}$ is the target vector of the RBF neural network corresponding to the input vector $x_{t}$, and mean square error γ of the RBF neural network can be expressed as:

(6) $$\gamma =\frac{1}{n}{\sum_{t=1}^{n}\left\|Y_{t}-y_{t}\right\|}^{2}.$$

Therefore, it is only necessary to learn and train three parameters of the RBF neural network so that the RBF neural network is able to establish the mapping relationship between the input vectors and the target vectors. In other words, the input sample set and the target sample set are used to train the RBF neural network, and the three parameters of the RBF neural network are constantly revised so that the output vectors of the RBF neural network can keep approaching the target vectors until the error expectation is reached.

The three parameters are the center of the radial basis function $c_{i}$, the extended constant of the center of the radial basis function $\sigma_{i}$, and the weight of the hidden layer to the output layer $w_{i}$. The self-organizing selection center method was used for training the RBF neural network in this paper. The method is detailed as follows:

(1) the k-means clustering algorithm was used to obtain cluster centers $c_{i}$, which were used as the center of the radial basis function of the RBF neural network;
(2) the Gauss function was used as the radial basis function, and the extended constant $\sigma_{i}$ can be calculated from the following relation:
  (7) $$\sigma_{i}=\frac{d_{\max}}{\sqrt{2N}}\left(i=1,2,\cdots ,N\right)$$
  where N is the number of the hidden nodes and $d_{\max}$ is the maximum distance between the selected cluster centers;
(3) the weights of the hidden layer to the output layer $w_{i}$ were calculated by using the least square method.

### 3.2. Simulation

The RBF neural network model was established in MATLAB, where the input layer had three nodes, and the output layer had one node. The number of the nodes of the hidden layer was determined by the mean square error γ of the RBF neural network. The smaller the γ is, the higher the precision of the model is, and the more nodes of the hidden layer are required. That is, if the error expectation is not reached, the number of the nodes of the hidden layer should be increased in the program. The radial basis function of the hidden layer was Gaussian function, and the training algorithm was the K-means clustering algorithm.

The sample data (shown in Figure 12) were used for training the RBF neural network. The resistances of the sixteen piezoresistive properties curves and the position coordinates X and Y of the sixteen small squares of the Velostat film were used as the three input vectors of the RBF neural network. The forces applied to the sixteen small squares of the Velostat film were used as the target vector of the RBF neural network. The input vectors and target vectors were used for training the RBF neural network so that the trained network was able to establish the mapping relationship between the input vectors (including the resistances and the position coordinates X and Y) and the target vector (including the force applied).

A rectangular coordinate system was established in the Velostat film, as shown in Figure 11. The position coordinates of the center of the sixteen small squares, $\left\{x_{i},y_{i}\right\}$, where i=1,⋯,16, were used as the two input vectors. The resistance of the sixteen piezoresistive properties curves $R_{ij}$, where i=1,⋯,16; j=1,⋯,8, were used as the third input vector. The force applied to the Velostat film $F_{i,j}$, where i=1,⋯,16; j=1,⋯,8, were used as the target vector.

In order to make the neural network converge better, the input vectors and target vector should be normalized first by the following formula:

(8) $$\{\begin{array}{c} {\bar{R}}_{ij}=\frac{R_{ij}-R_{i,\min}}{R_{i,\max}-R_{i,\min}}\\ {\bar{F}}_{ij}=\frac{F_{\max}-F_{ij}}{F_{\max}-F_{\min}} \end{array}(i=1,2,\cdots ,16;j=1,2,\cdots ,8)$$

where ${\bar{R}}_{ij}$, ${\bar{F}}_{i}$ are the normalized vectors, $R_{i,\max}$, $R_{i,\min}$ are the maximum and the minimum sample data of the resistance of the *i*th small square, and $F_{\max}$, $F_{\min}$ are the maximum and the minimum force applied to the Velostat film.

**Figure 14:**
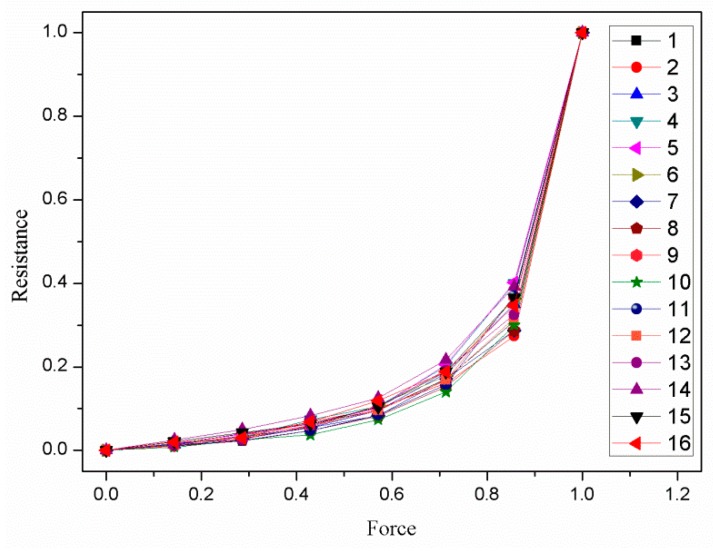
The normalized sample data.

The normalized sample data is shown in Figure 14.

**Figure 15:**
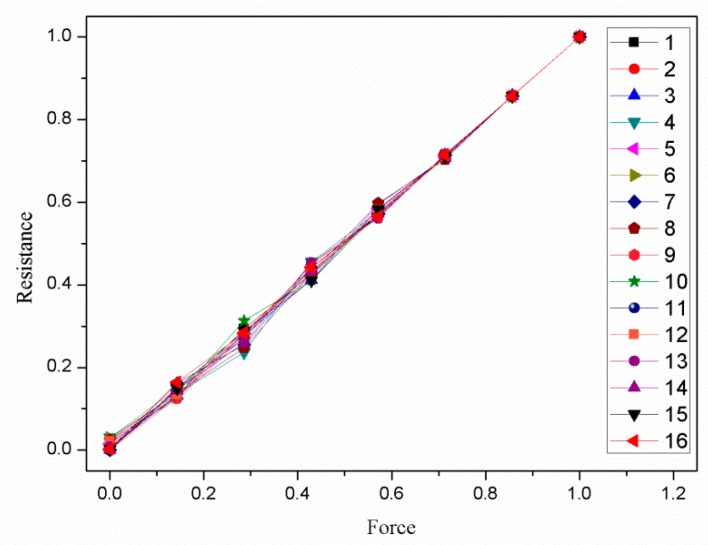
The output of the trained RBF neural network.

Finally, the normalized input vectors and target vector were used for training the RBF neural network. Let the mean square error γ=0.001. Then, the parameters of the RBF neural network were constantly revised by training so that the output vectors of the trained RBF neural network could keep approaching the target vector until the error expectation was reached. The output of the trained RBF neural network was shown in Figure 15.

Comparing Figure 14 and Figure 15, the relationship between the resistance and the force were linearized effectively, and the consistency of the piezoresistive properties curves of the sixteen small squares was improved. The maximum nonlinear error was reduced from 58.3% to 4.5%. The maximum relative standard deviation was reduced from 29.1% to 8.6%. It is worth noting that the consistency of the piezoresistive properties curves can be further improved by reducing the mean square error.

Therefore, on the premise of obtaining the contact point position, the RBF neural network algorithm is suitable to be used for training and calibrating the tactile sensor for the purpose of detecting the contact force.

## 4. System Application

**Figure 16:**
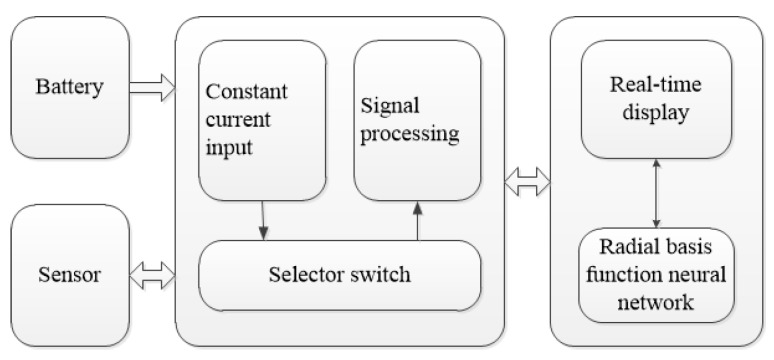
Structure diagram of the signal processing system.

Based on the PIC18F25K80 chip, we built the signal processing circuit of the tactile sensor. The signal processing circuit used a 6 V battery as an external power supply that made the use of the sensor more convenient. The display interface of the sensor was developed by LABVIEW. The tactile sensor system could display the position and force of the contact point in real time by directly calling the trained RBF neural network. It is worth mentioning that the sensor used a DC constant current, and the input current was only 10 mA. Therefore, the power consumption of the sensor system was very small. The signal processing system of the tactile sensor is shown in Figure 16.

It should be noted out that the contact area of the tactile sensor has a great influence on the contact force detection. Considering that people use finger touch sensors for human–computer interaction, the contact area is about 15 × 15 mm, so we used a rubber block with 15 × 15 mm dimensions to simulate a human finger to calibrate the tactile sensor for detecting contact force.

First, the rubber block was placed at the center of the sixteen small squares of the sensor sample, and a force of 3 to 15 N was applied to the rubber block by the ZQ-20B-1 spring testing machine. The output resistances between electrodes C and E of the tactile sensor and the position coordinates of the contact point were used as the three input vectors of the RBF neural network. The force applied to the rubber block was used as the target vector of the RBF neural network. Next, the input vectors and target vector were normalized and used for training the RBF neural network. Finally, the trained RBF neural network was stored in a MATLAB file for the tactile sensor system to call. Thus, the calibration of the contact force detection of the tactile sensor was completed.

**Figure 17:**
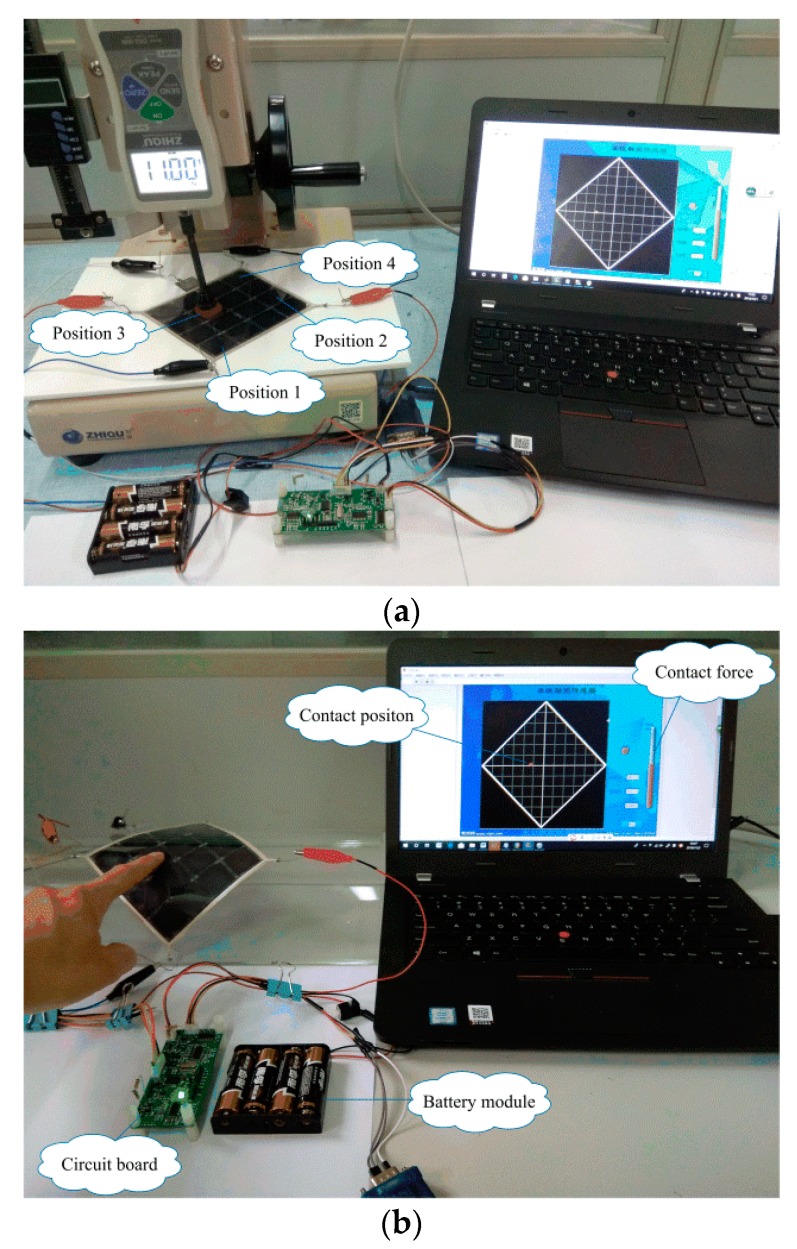
The test device. (**a**) The tactile sensor was used to detect the contact position and force; (**b**) using a finger to touch the tactile sensor when it was pasted onto the curved panel.

**Figure 18:**
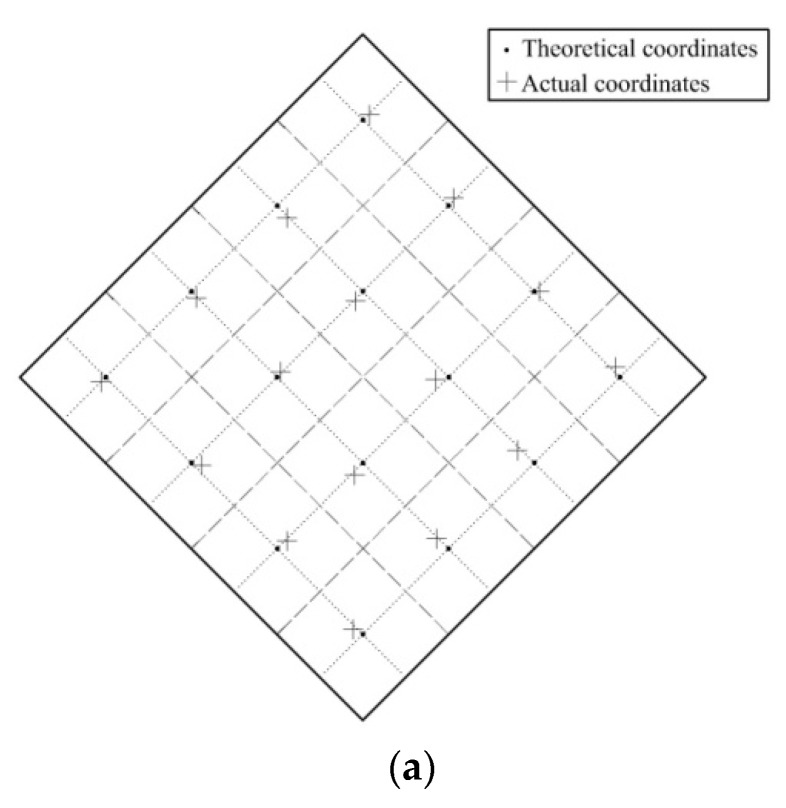

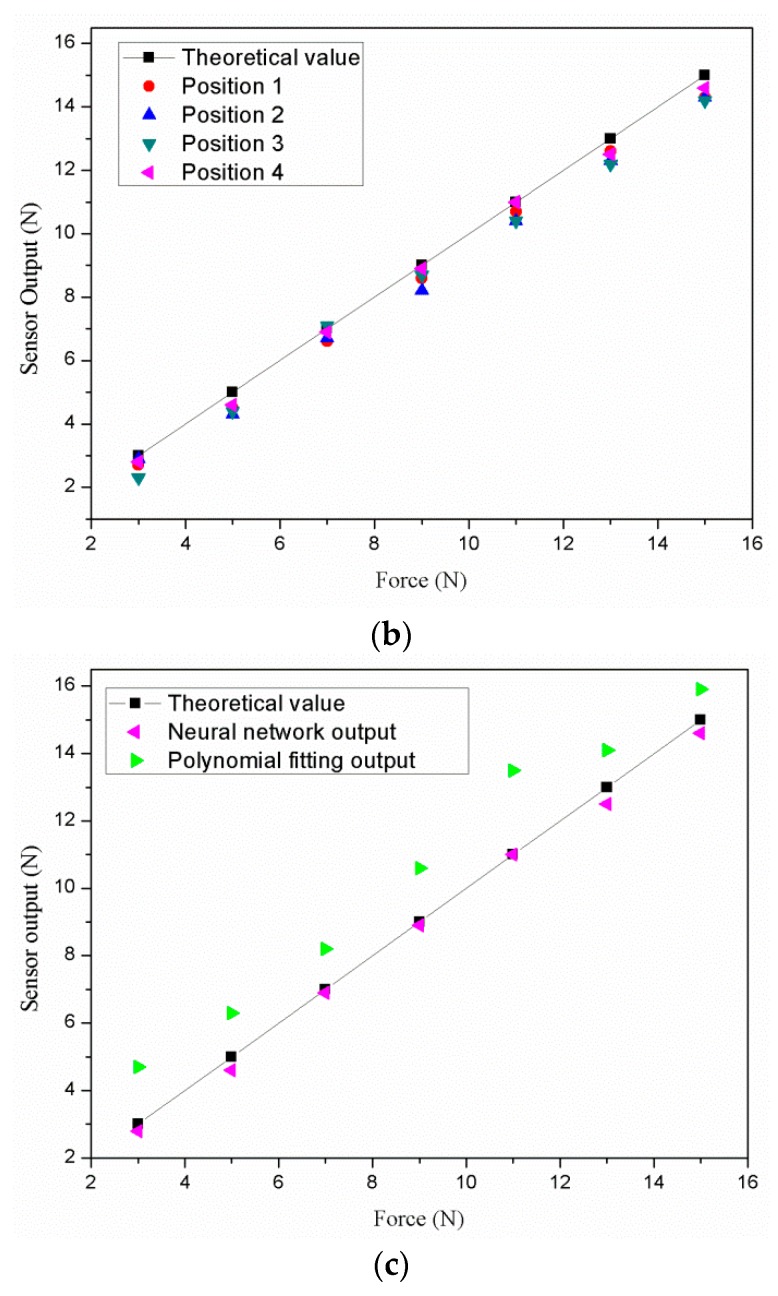
The results of the experiments. (**a**) The result of contact position detection; (**b**) the result of contact force detection; (**c**) comparison of the RBF neural network output with the polynomial fitting output.

The experiments detecting position and force were carried out using the calibrated tactile sensor system, and the test device is shown in Figure 17a. First, contact position detection was carried out. The rubber block was placed at the center of the sixteen small squares of the sensor, and a 7 N force was applied to the rubber block by the ZQ-20B-1 spring testing machine. The contact position of the tactile sensor system output was shown in Figure 18a. Contact force detection was then carried out. Four dispersed small squares (shown in Figure 17a) were selected to be subjected to be applied forces of 3–15 N. The contact force of the tactile sensor system output was shown in Figure 18b.

In addition, by the means of the polynomial curve fit, the average of the sixteen piezoresistive properties curves (shown in Figure 12) was used to calibrate the sensor for detecting the contact force. The fourth position (shown in Figure 17a) of the tactile sensor was tested by the two algorithms. The sensor outputs were shown in Figure 18c.

Furthermore, the sensor was pasted onto the curved panel with a radius of curvature of 200 mm, as shown in Figure 17b and video S1 (Supplementary Materials). When we used the finger to touch the sensor, the tactile sensor system could display the contact position and force in real time.

The experiment showed that the maximum measurement error of the contact force detection was not more than 5 mm. The main reasons for the error were that the silver paste circuit featured a degree of unevenness, and the voltage drop distribution of the circuit was not uniform, resulting in non-uniform gradient distribution of the potential in the conductive plane. The maximum measurement error of the contact force detection was not more than 1 N. The main reasons for the error were: (1) the force applied by the spring testing machine was not accurate enough. (2) The Velostat film is a viscoelastic material with creep properties, and its internal resistance will change with time when it is applied with a constant force. We used the resistance of the Velostat film after it was applied the force for about 1 s as the sample data. However the sampling time was not accurate enough. (3) The RBF neural network model itself has errors. In comparison of the RBF neural network algorithm with the polynomial fitting algorithm, the maximum error of the contact force detection was reduced from 2.7 N to 0.5 N.

## 5. Conclusions

A novel flexible tactile sensor that can detect the contact position and force was proposed in this paper. The sensor was composed of three thin-film layers. It had a simple structure, good flexibility, small thickness and only five lead wires. In order to solve the inconsistency and nonlinearity of the piezoresistive properties for large areas, an RBF neural network was used as a contact force detection algorithm. The sensor sample was made. Experiments on the contact position and force detection of the sensor sample were carried out. The experimental results showed that the principal functioning of the sensor was feasible. The tactile sensor was capable of detecting position and force for a single point and could easily be used to cover the surfaces of objects. The RBF neural network algorithm is very effective for correcting the inconsistency and nonlinearity of the tactile sensor. The maximum relative standard deviation was reduced from 29.1% to 8.6%. The maximum nonlinear error was reduced from 58.3% to 4.5%. Compared with the polynomial fitting algorithm, the maximum error of the contact force detection was reduced from 2.7 N to 0.5 N. The sensor can provide the necessary information to support safe human–robot interactions.

## Supplementary Materials

The following are available online at http://www.mdpi.com/1424-8220/19/1/27/s1, Video S1: Experimental video.
